# Wearable Inertial Sensors for Daily Activity Analysis Based on Adam Optimization and the Maximum Entropy Markov Model

**DOI:** 10.3390/e22050579

**Published:** 2020-05-20

**Authors:** Sheikh Badar ud din Tahir, Ahmad Jalal, Kibum Kim

**Affiliations:** 1 Department of Computer Science, Air University, Islamabad 44000, Pakistan; 181483@students.au.edu.pk (S.B.u.d.T.); ahmadjalal@mail.au.edu.pk (A.J.); 2 Department of Human-Computer Interaction, Hanyang University, Ansan 15588, Korea

**Keywords:** Adam optimization, accelerometer and gyroscope sensors, inertial sensors, multi-fused features, maximum entropy Markov model

## Abstract

Advancements in wearable sensors technologies provide prominent effects in the daily life activities of humans. These wearable sensors are gaining more awareness in healthcare for the elderly to ensure their independent living and to improve their comfort. In this paper, we present a human activity recognition model that acquires signal data from motion node sensors including inertial sensors, i.e., gyroscopes and accelerometers. First, the inertial data is processed via multiple filters such as Savitzky–Golay, median and hampel filters to examine lower/upper cutoff frequency behaviors. Second, it extracts a multifused model for statistical, wavelet and binary features to maximize the occurrence of optimal feature values. Then, adaptive moment estimation (Adam) and AdaDelta are introduced in a feature optimization phase to adopt learning rate patterns. These optimized patterns are further processed by the maximum entropy Markov model (MEMM) for empirical expectation and highest entropy, which measure signal variances for outperformed accuracy results. Our model was experimentally evaluated on University of Southern California Human Activity Dataset (USC-HAD) as a benchmark dataset and on an Intelligent Mediasporting behavior (IMSB), which is a new self-annotated sports dataset. For evaluation, we used the “leave-one-out” cross validation scheme and the results outperformed existing well-known statistical state-of-the-art methods by achieving an improved recognition accuracy of 91.25%, 93.66% and 90.91% when compared with USC-HAD, IMSB, and Mhealth datasets, respectively. The proposed system should be applicable to man–machine interface domains, such as health exercises, robot learning, interactive games and pattern-based surveillance.

## 1. Introduction

Nowadays, rapid development in the area of wearable sensors has revolutionized the monitoring of human life logs in indoor/outdoor environments. These advancements enabled us to develop sophisticated sensors, which are attached safely on human body and monitor behavioral patterns. In addition, these enhancements have developed personalized environments which has led to improving the living standard of humans. However, the sensors for life log monitoring still have challenges in recognizing activities with minimal contextual information. Similarly, several limitations such as inconsistent human motions, resting, unconsciousness and overflow breathing reflect on sensors’ inaccurate human activities detection and cause difficulties in recognizing activities with complex postures. 

Currently, wearable sensors have a wide range of real-world applications of human activity recognition (HAR). The applications include security surveillance systems, healthcare monitoring, sports assistance, interactive 3D games and smart homes [1]. In security, uncertain event detection applications are examined using HAR systems to take safety measures against violence activities (i.e., fighting, falling, and strikes) in the surrounding area. In healthcare monitoring, it is possible to analyze a patient’s heart rate, body motion, brain activity and other critical health data that become suitable to assist them in usual or unusual patient’s behavior. On the other hand, in the rehabilitation process, patients can easily monitor their health fitness and medication routines. In sports assistance, these wearable sensors provide velocity tracking for physical trainings and sweat rate [2] in order to make them conduct exercises more effectively. In interactive 3D games, body part movements are controlled by wearable sensors and physical exergames are playable in indoor environment, while in smart homes, long-range distance care is provided, such as children day-care and elderly activities monitoring.

Considering the wearable sensor-based technologies for HAR, a wide range of sensors have been integrated to get distinctness in the acquisition of cue understanding. Among them, a few wearable sensors like accelerometers, gyroscopes and magnetometers make it feasible to detect multiple aspects of human life and to measure position changes, angular rotation and body movements in 3-dimensional space [3,4,5]. However, despite such substantial amounts of information provided by wearable sensors, there are still some HAR challenges [6,7] that face the unresolved issues and lack the capability of giving perfect HAR results. 

In this paper, we propose a novel multifused wearable HAR system that overcomes the complexity of human life log routines by measuring the changes of body position and orientation. For the inertial (i.e., accelerometers and gyroscopes) filtered data, we have adapted all the three main approaches as statistical features (i.e., median, variance, etc.), frequency features, and wavelet transform features. However, as a large dimension of features increases the computational HAR complexity, we use adaptive moment estimation (Adam) and AdaDelta optimization algorithms to properly discriminate among various activity classes. Finally, the maximum entropy Markov model is embodied in the model to measure empirical expectation and highest entropy of various human activities to obtain significant accuracy. As a performance evaluation, the comparison between the proposed classifier and conventional classifiers was performed. We applied our model to the IM-Sporting Behaviors (IMSB) dataset, which is based on different signal patterns of sport activities. Simultaneously, we utilized the proposed model for two public inertial sensor-based datasets called the University of Southern California human activity dataset (USC-HAD) and the Mhealth human dataset. The main contributions of this paper are as follows:

- We proposed multifeature extraction methods having both time domain and frequency domain features of varied signal patterns.
- For complex human activity patterns of sports and daily life living, we designed an Adam optimization-based maximum entropy Markov model that provided contextual information as well as classifying behaviors.
- In addition, comprehensive evaluation was performed on two public benchmark datasets as well as one self-annotated dataset, which achieved significant results when compared with other state-of-the-art methods.

The remaining parts of the paper are organized as follows: In Section 2, we review the related work of two main categories of human activity analysis. Section 3 addresses the proposed HAR model with the integration of multifused methods, Adam optimization, and the maximum entropy Markov model. Section 4 presents the experimental setup and the results of the comparison with existing well-known statistical state-of-the-art methods. Finally, Section 5 discusses conclusion and future directions.

## 2. Related Work

A significant amount of research is undergoing for the development of HAR via multiple categories of sensors, such as video sensors and body-worn inertial sensors. Here, we comprehensively divide the related work into two parts, video sensor based HAR analysis and wearable sensors based activity analysis.

### 2.1. Video Sensor-Based HAR Analysis

In vision sensors, video cameras are fixed at surveillance locations to perform automated inspections of human activities. In recent years, these sensors have also become widely used in healthcare and fitness industries for researchers to improve the practices of user authentication [8,9]. Htike et al. [10] proposed a posture-based HAR system using one static camera. For video surveillance, their model is distributed into two stages; training and evolution stages. These stages were further implemented by four different classifiers, namely, a feed-forward neural network, K-means, multilayer perceptron, and fuzzy C-means. In [11], Jalal et al. designed a depth video-based translation and scaling invariant HAR model with the combination of a hidden Markov model (HMM). In their work, the invariant features are computed by different transforms such as R transformation and Radon transformation. Furthermore, for classification, they used HMM and principal component analysis (PCA) to recognize multiple human activities.

Babiker et al. [12] proposed series of preprocessing operations which include background subtraction, binarization, and morphological techniques. In addition, for classification of activities, a robust neural network model-based multilayer feed forward perceptron network was used. In [13], Zhou et al. developed an adaptive learning method to determine the physical location and motion speed of a human from a single camera view without estimating (i.e., intrinsic and extrinsic parameters) of camera calibration in indoor environments. Additionally, to recognize the human activities, hierarchical decision tree and size reduction methods were used.

### 2.2. Wearable Sensor-Based Activity Analysis

In recent decades, a significant amount of HAR research work has been mainly adapted through visual information [14]. However, due to several limitations in HAR such as long-range human movements and illumination changes of vision sensors, wearable-sensor technology has been gaining attentions as a new solution among researchers in the community [15]. In addition, the demands for understanding and monitoring human life log activities via wearable sensors have grown incrementally. In [16], Roy et al. proposed a hybrid method for recognizing daily living activities in smart environment using body-worn sensors and ambient sensors. This work focused on spatiotemporal constraints that improved the accuracy and reduced the overhead computation of the HAR system. Nweke et al. [17] presented analysis of human activity detection and monitoring by multisensor fusion via an accelerometer and a gyroscope. They attached multiple sensors on different body locations (i.e., wrist, chest, ankle and hip) and obtained good results via random forest (RF) and voting significant schemes.

In [18], Zebin et al. used inertial sensors on five different sensor locations on the lower body. These different body locations were selected to classify human activities more precisely than in HAR. The authors systematically proposed a feature learning method to automate feature learning from raw input using convolutional neural networks (CNN). Zhu et al. [19] developed a multisensor fusion network using two inertial sensors, which were attached one on the foot and the other on the waist of a human subject. They combined the multisensor fusion method with HMM and neural networks to reduce the computational complexity and to obtain better accuracy. 

In spite of previous HAR research, there are still challenges in dynamic movement, multisensor computations and precise signal data acquisition. Therefore, we suggest a novel methodology for HAR in this paper.

## 3. Designed Framework for Wearable HAR

**Figure 1:**
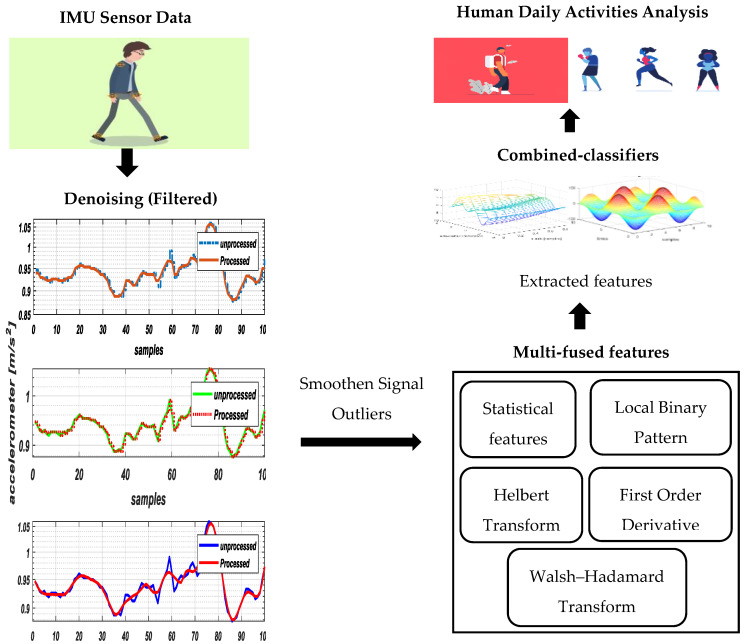
Flow architecture of proposed human activity recognition model.

Initially, the purposed HAR model acquire data as input from inertial measurement unit (IMU) sensors having 3-axis signal values of accelerometers, gyroscopes and magnetometers. These signals pass through stages of perusal and standardized procedures to realize the taste of reasonable classification. Firstly, raw signals are divided into frame size (i.e., 50 ms) via fixed-sliding window analysis. These signals involve multiple filters such as median, Savitzky–Golay and hampel to refine themselves by eliminating small saw tooth waves from the data. Secondly, in the signal normalization phase, we ensure other denoising effects (i.e., short-term fluctuation, maximum noise removal, etc.) that smoothen signal outliers. Thirdly, we propose multifused features from different domains including statistics, frequency and time, which are quantized via codebook generation for proper symbol selection. Finally, in the wearable HAR classification module, a novel combined classifier of Adam optimization and the maximum entropy Markov model is implemented on the optimal feature sets in order to recognize different activities. The schematic diagram of our complete model is shown in Figure 1. 

### 3.1. Data Acquisition and Denoising

**Figure 2:**
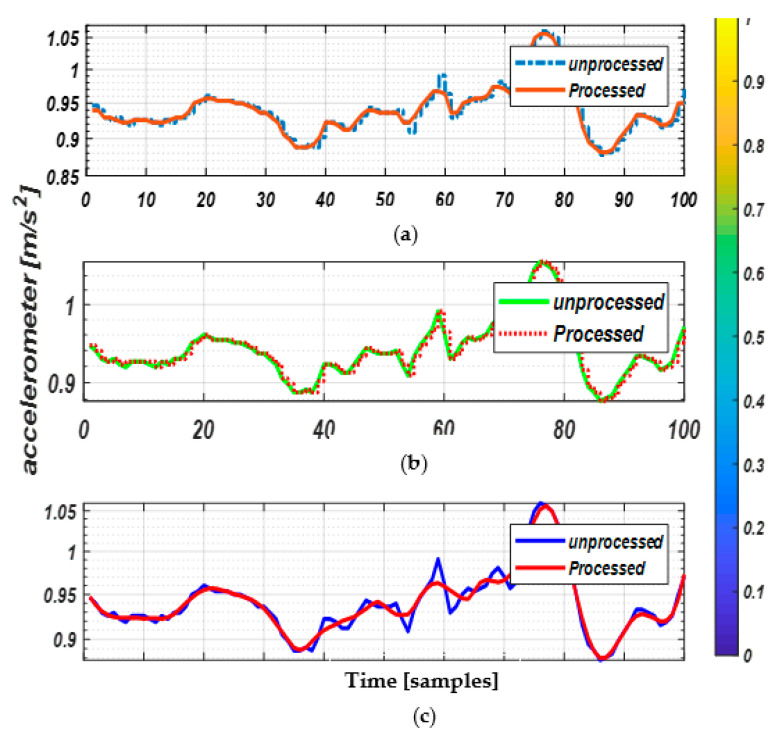
Signal Preprocessing. Inertial sensors with unfiltered (unprocessed) and filtered (processed) signals of correct walking activity via (**a**) Savitzky–Golay, (**b**) hampel and (**c**) median filters on the University of Southern California Human Activity Dataset (USC-HAD) dataset.

As IMU sensors are highly sensitive to even minor amounts of random noise, any intentional/unintentional change may cause irrelevancy among signal values and may further alter signal shapes, which badly affects the feature extraction phase. For this reason, three different filtration techniques, i.e., Savitzky–Golay, median and hampel filters are applied to the datasets to eliminate the noise associated with the inertial signals. In Savitzky–Golay (Figure 2a), a set of digital data points are computed to smoothen the raw data and to increase the precision of data without misleading the signal outliers. Similarly, the hampel filter (Figure 2b) detects and removes the random outliers of the raw signals, whereas, the median filter (Figure 2c) acts as a nonlinear approach that eliminates the impulsive type of noise and restores the processed signals to nearly normal motions. Based on these signal acquisition, we adopted the median filter which provided better results when compared to the other filters’ impulsive types of noise in all three datasets.

### 3.2. Windowing Selection

In order to maximize the recognition accuracy, window selection has to be chosen to obtain more contextual information [20]. For window selection, we studied different windowing strategies that have been adopted by many researchers. In inertial-based sensor work, most researchers windowed their signals in the segment ranges of 4–5 seconds to analyze the daily activities of humans. The cyclic motion patterns in USC-HAD, Intelligent Media sporting behavior (IMSB) and Mhealth datasets make it easier to understand and to recognize the windowing selection mechanism with the fixed-sliding [21]. 

### 3.3. Feature Extraction Methods

After signals selection, we discuss our proposed multifused feature model in which a detailed description of three major domains (i.e., statistical features, frequency and wavelet) are combined together for validation. Statistical features are measured by clear-cut segregation of signal values and as such are computationally less intensive, whereas frequency domain features mainly focus on a periodic structure of the signal, such as spectral entropy and the Helbert transform. In addition, wavelet features are commonly used to find absolute patterns of the signal, i.e., the Walsh–Hadamard transform. Algorithm 1 defines the multifused features model.
**Algorithm 1: Inertial Signal Features Computation****Input**: Acc = Accelerometer (x,y,z), Gyr = Gyroscope (x,y,z) and SR = Sampling Rate (100 Hz)**Output**: Multifused feature vectors $\left(u_{1},u_{2},u_{3}\ldots \ldots u_{n}\right)$featurevectors← []samplesignal← GetSampleSignal()  Overlap ← GetOverlappingTime()**Procedure** HAR(Acc,Gyr,SR)MultiFusedVector← []DenoiseData ← MedianFilter(Acc,Gyr)SampledData(DenoiseData,SR) While exit condition not satisfied do[min, max, mean, variance]← ExtractStatisticalFeatures(sample data)[LBPFeatures]← ExtractLocalBinaryPatternFeatures(sample signal)[WHT, CZT, HT]← ExtractWaveletFeatures(sampledsignal)MultiFusedVector← [ min, max, mean, variance, LBP, WHT, CZT, HT]Return MultiFusedVector

#### 3.3.1. Statistical Features

**Figure 3:**
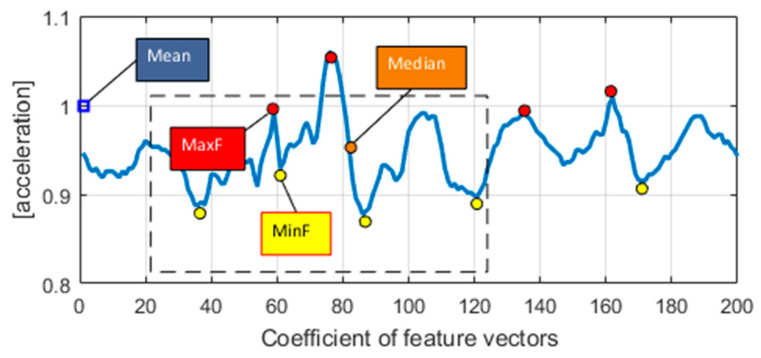
1D vector plot of statistical features of the walking forward activity log using the USC-HAD dataset.

The statistical features S(V_stat_) reflect the average, middle, squared deviation and max/min values of sample *i* signal in each frame. These features hold a major factor to examine the overall changes that are explored as a response for each activity of n as

(1) $$S(V_{\mathrm{stat}})=\overset{n}{\underset{i=1}{\sum }}\frac{c_{i}}{n},\;\frac{\sum_{i=1}^{n}{\left(Z-\bar{Z}\right)}^{2}}{n-1},\;\min\left(\mathrm{signal}\right)\left(M_{i}\right),\;\max\left(\mathrm{signal}\right)\left(X_{i}\right)$$

where n is sampled data size, c is the coefficients in the feature vector, Z is the value of first vector and $\bar{Z}$ is the mean of all sampled data. Figure 3 shows a 1D plot with a combination of different statistical features of the walking forward activity log using the USC-HAD dataset.

#### 3.3.2. Chirp Z-Transform

**Figure 4:**
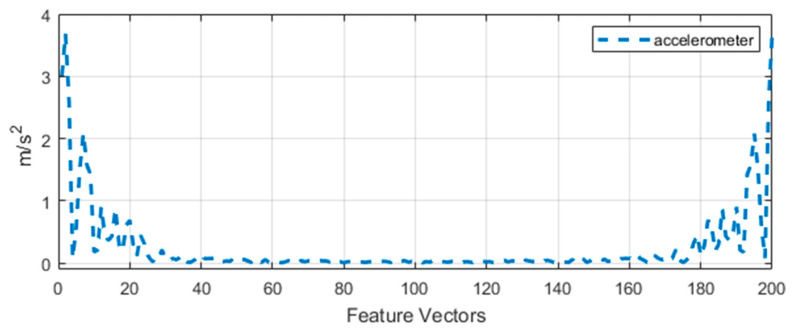
Accelerometer signal representation via chirp z-transform of the walking forward signal.

In frequency domain features, the chirp z-transform (CZT) is used as a high-speed convolution algorithm that evaluates the z-transform of a signal [22]. The functions are viewed as polynomials with poles as roots and zeros, where poles are the peak energy spectrum concentration and zeros are modeled on frequency spectrum troughs (Figure 4). It helps to estimate the transfer function of a system by an accurate number of zeros. Poles in the system cause a finer bandwidth dimensions and efficient reduction of the transfer function in polynomials ratios. It is defined as

(2) $$X_{k}=\overset{N}{\underset{i=0}{\sum }}x\left(n\right)z_{k}^{-n}$$

(3) $$z_{k}=A.W^{-k},\;k=0,1,\ldots B-1$$

where x(n) is the original signal, z is the arbitrary complex number up to n number of points. A is the starting point of the complex number and W is the complex ratio up to the points of k. 

#### 3.3.3. Helbert Transform

**Figure 5:**
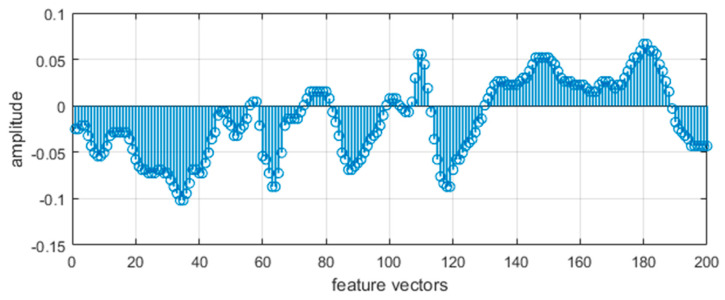
Hilbert transform features depicted for *x* components of the walking forward signal.

During Helbert transform, we identified the minimum level of frequency retained by calculating the Fourier transform of the given signal *a*(*t*) to discard the negative frequencies and to double the magnitude of positive values [23]. These outputs become the complex-valued signals in which imaginary and real part values form a Hilbert transform pair, as shown in Figure 5. This pair acts as a specific linear operator, which gives the Hilbert space of real eigenstate values of

(4) $$H\left(a\right)\left(t\right)=\frac{1}{\pi }\int_{-\infty }^{\infty }\frac{x\left(a\right)}{t-a}\mathrm{da}$$

where x(a) is the signal which has the amplitude spectrum and autocorrelation function. x(a) is the real variable signal/real data sequence. The input data is zero-padded or truncated to length t − a.

#### 3.3.4. D Local Binary Pattern (1D LBP)

**Figure 6:**
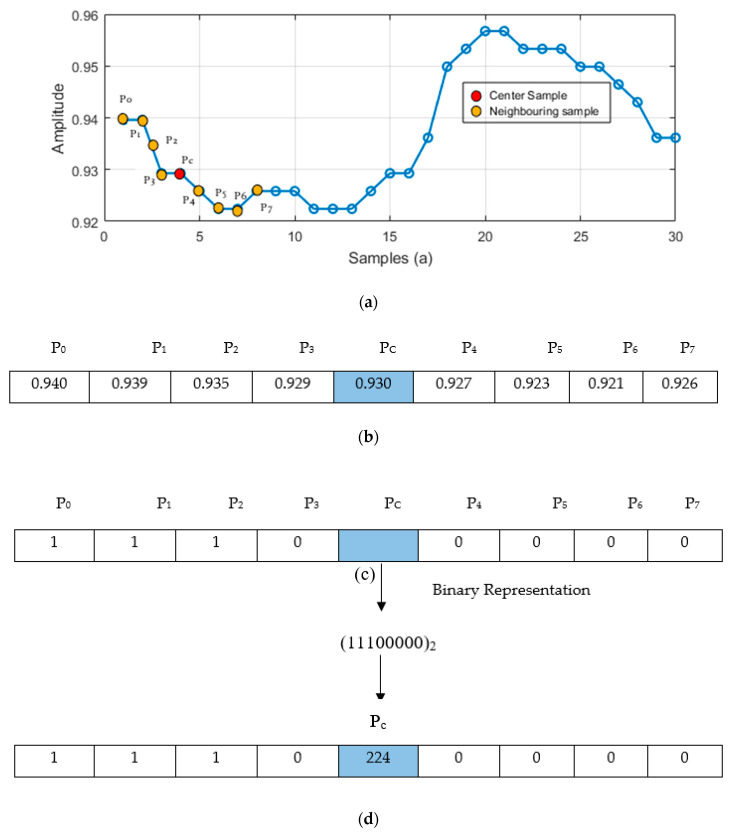
Local binary pattern (LBP) applied using signal data. (**a**) Segment of inertial signal sample, (**b**) sample values of associate signal, (**c**) middle value P_c_ as threshold for associate values P_o_, P_1_, P_2_, …, P_7_ and (**d**) produced LBP code converted into decimal representation.

1D LPB is statistically used as a nonparametric operator which defines the number of counts for each change in the inertial sensors signals that exceed the threshold. For each data sample, a binary code produced and found precise variations in the processed inertial signals [24]. In this algorithm, the middle sample is selected as a threshold value and the other values are compared against the particular threshold. If the values are smaller than the threshold value, it is set to 0, and vice versa (Figure 6). The formation of 1D LBP using the inertial signal is defined as

(5) $$1\mathrm{DLBP}\left(x,y,z\right)=\overset{n}{\underset{i=0}{\sum }}S_{\mathrm{iner}}\left(t\right)2^{i}$$

where $S_{\mathrm{iner}}\{\begin{array}{ll} 1, & t\geq \mathrm{threshold}\\ 0, & t<\mathrm{threshold} \end{array}$.

#### 3.3.5. Walsh–Hadamard Transform

**Figure 7:**
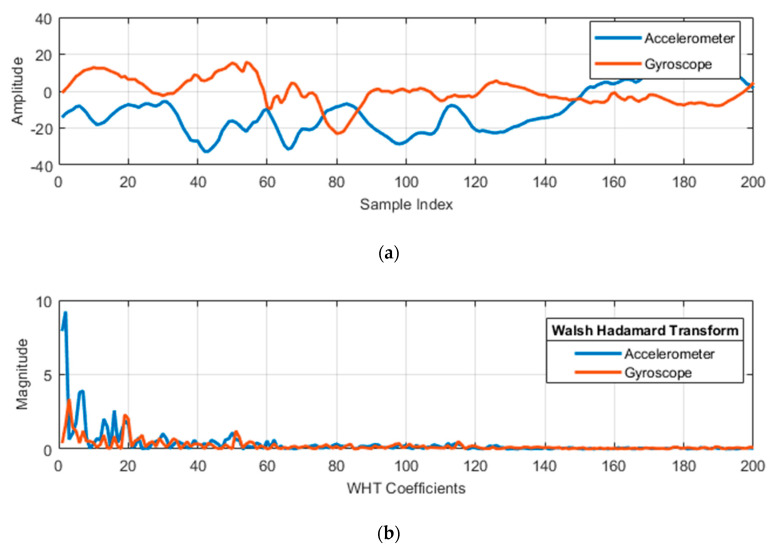
1-D Walsh–Hadamard transform (WHT) as (**a**) a WHT signal feature and (**b**) magnitude of WHT coefficients of the walking forward activity.

Walsh–Hadamard transform (WHT) is used as an orthogonal transformation that splits our inertial signal into a set of signals [25]. Then, it finds dense property (i.e., energy of these signals), which deals with the real numbers, helps to minimize the computational costs [26] and produces a more robust set of features. Figure 7 represents the sensor fusion of an accelerometer and gyroscope with motion patterns of walking forward activity via WHT, respectively. The discrete Walsh–Hadamard transform (DWHT) of a vector is represented by

(6) $$X_{w}\left(k\right)=\overset{N-1}{\underset{n=0}{\sum }}x\left(n\right)\overset{M-1}{\underset{i=0}{\prod }}{\left(-1\right)}^{n_{i}K_{M-1-i}},\;k=0,\;1,\ldots ,N-1$$

where N is the number of samples of the vector data and M = $\log_{2}N$.

#### 3.3.6. First Order Derivatives

**Figure 8:**
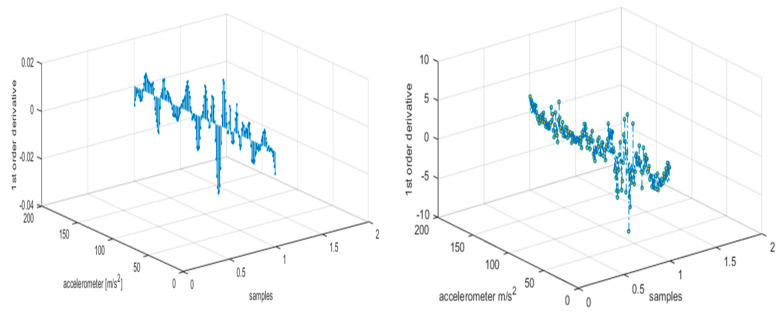
First order derivation representation of walking forward and jumping up activities using the USC-HAD dataset.

During first order derivation, we calculated the rate of change of inertial sensors’ coordinates (i.e., X, Y, Z) and found the direction of a signal, which measures the slope of the tangent to the signal and explores the instantaneous rate of change in a signal (See Figure 8). This implies how rapidly the adjacent points change their positions over time. They are computed as

(7) $$X_{i}=\frac{X_{i+1}-Y_{i}}{\Delta Y},\;Y_{i}=\frac{Y_{i+1}-Y_{i}}{\Delta Z},{\;Z}_{i}=\frac{Z_{i+1}-Z_{i}}{\Delta X},\;\;\mathrm{where}\;I=1,\;2\ldots .\;n-1$$

## 4. Combined Classifiers

To enhance the accuracy performance of the multifused features model, we used the combined classifiers strategy in which optimization techniques work as preclassifiers. For wearable HAR classification, we applied the maximum entropy Markov model along with Adam and AdaDelta optimizations techniques.

### 4.1. Adam Optimization Algorithm

**Figure 9:**
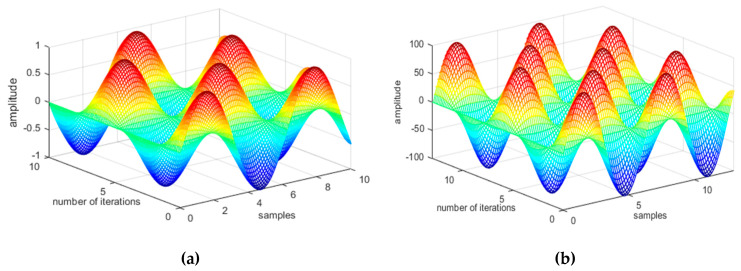
Adaptive moment estimation (Adam) optimization algorithms with adaptive learning of (**a**) walking forward and (**b**) running forward activities using the USC-HAD dataset.

Optimization algorithms are used as a progressive set of routines that calculates adaptive learning rate and finds the closest optimal solutions for problems with a confused set of operations. Adaptive moment estimation (Adam) [27] is among the essential strategies of optimization algorithms that compute individual adaptive learning rates based on the first and the second moments of the gradients. In our case, we trained the model with a learning rate of 0.00005. In addition, it computes the finest properties of both algorithms, named RmsProp and AdaGrad. RmsProp provides the average of recent magnitudes of the inertial signals gradients and AdaGrad deals with sparse gradients with uncentered variance. Both these algorithms are formulated as

(8) $$m_{t}={\beta m}_{t}-1+\left(1-{\mathrm{beta}}_{1}\right)g_{t}$$

(9) $$v_{t}=\beta_{2}v_{t}-1+\left(1-{\mathrm{beta}}_{2}\right)g^{2}{}_{t}$$

where $m_{t}$ and $v_{t}$ are estimates of the first moment of the mean and the second moment of the uncentered variance, respectively. Figure 9 shows a 3D plot of Adam optimization values of walking forward and running forward activities.

### 4.2. AdaDelta Optimization Algorithm

**Figure 10:**
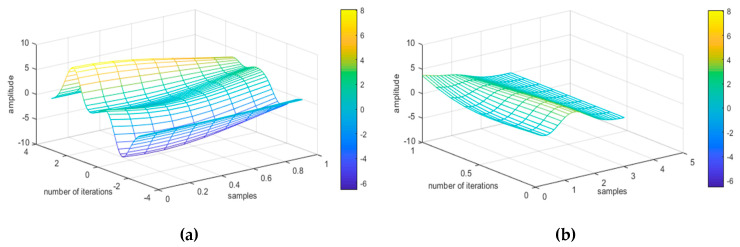
AdaDelta optimization algorithm with adaptive learning of (**a**) elevator down and (**b**) elevator up activities using the USC-HAD dataset.

In the case of the AdaDelta optimization algorithm [28], we adapted learning rates based on a moving window of gradient descent updates. At instances, it continues learning updates and tunes parameters to obtain maximum possible learning values. On the other hand, the sum of gradients is recursively defined as the running average E[g^2^]_t_ at current time step, and also depends on the previous average and the current gradient

(10) $$E{\left[g^{2}\right]}_{t}=\mathrm{rE}{\left[g^{2}\right]}_{t-1}+\left(1-r\right)g_{t}^{2}$$

where r is the fraction similar to the momentum term. Figure 10 shows the AdaDelta results of different activities using the USC-HAD dataset.

### 4.3. The Maximum Entropy Markov Model 

After feature vector optimization, we tested our proposed model against two challenging datasets that contain activities of multiple classes. For a multiclass problem, we aimed to measure empirical expectation and highest entropy of different activities using the maximum entropy Markov model (MEMM). The idea behind the decision to use this model was to overcome the concept of the conventional hidden Markov model (HMM) [29] framework in which observation and transition functions are replaced by an individual function $P(s|s^{\prime },\;o).$ Thus, the present observation depends on the present state. 

**Figure 11:**
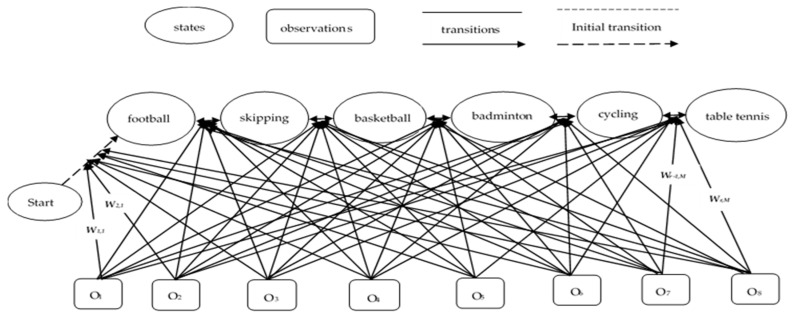
Maximum entropy Markov model algorithm applied to six different activities of the Intelligent Media sporting behavior (IMSB) dataset.

In contrast, instead of relying on its present states, MEMM can think of the observations as being associated with transition states. Initially, all observations are connected with state transitions instead of with states. It is defined as

(11) $$\alpha_{t+1}\left(s\right)=\underset{s^{\prime }}{\sum }\alpha_{t}\left(s^{\prime }\right)*P_{s^{\prime }}(s|o_{t+1})$$

where $\alpha_{t}\left(s\right)$ is the probability of states at given time t to the observation categorization. Then, it estimates probability distribution of the data that are dependable on certain constraints procured from the training data. Each constraint indicates some characteristics of the training data which predict values of all individual features in the learned distribution. In addition, it emits the tokens from the training data which determine the best set of observation features and are also able to solve the multinomial classification based on prediction problems. This classification model generalizes to find coefficients that match the breakdown of the dependent variable as

(12) $$P\left(S|O\right)=\overset{n}{\underset{r=1}{\prod }}P\left(O_{r}|q_{r}\right)*\overset{n}{\underset{r=1}{\prod }}P\left(S_{r}|S_{r-1}\right)$$

where S is the state sequence and O is the sequence of observations, i.e., O_1_, O_2_, …, O_n_. In order to maximize the conditional probability P, a set of observations is tagged with labels S_1_, S_2_, …, S_n_, as

(13) $$P\left(S_{1},\ldots ,S_{n}|O_{1},\ldots ,O_{n}\right)=\overset{n}{\underset{r=1}{\prod }}P\left(S_{r}|S_{r-1},O_{r}\right)$$

(14) $$\frac{1}{m_{s_{i}}}\sum_{r=1}^{m_{s_{i}}}f_{r}\left(o_{t_{r}},s_{t_{r}}\right)=\frac{1}{m_{s_{I}}}\sum_{r=1}^{m_{s_{i}}}\sum_{s\in S}P_{s^{\prime }}\left(s|o_{t_{r}}\right)f_{a}\left(o_{t_{r}},s\right)$$

where t_1_, t_2_, t_3_, …, $t_{r}$ are the time stamps that comprise of transition function $P_{s^{\prime }}.$

(15) $$P(s|s^{\prime },\;o)=\frac{1}{Z\left(o,s^{\prime }\right)}\exp\left(\underset{r}{\sum }w_{r}f_{r}\left(o,s^{\prime }\right)\right)$$

where $w_{r}$ is the weight to be learned and is associated with the feature $f_{r}\left(o,s^{\prime }\right)$ acting as the categorical feature functions also known as real-valued, and $Z\left(o,s^{\prime }\right)$ is the normalizing term ensuring the matrix sum. Figure 11 describes the overall flow of MEMM applied to six different activities of the IMSB dataset.

## 5. Experimental Results and Analysis

### 5.1. Experimental Setting

To evaluate the training/testing performance of the proposed model, we used the “leave-one-out” cross validation method on three benchmark datasets named USC-HAD (ACM, Pittsburgh, PA, USA), IMSB (Islamabad, Pakistan) and Mhealth (IWAAL, Belfast, Northern Ireland). These datasets include multiple activities taken in different environments, i.e., public areas, sports fields and indoor-outdoor locations. Inertial sensors (i.e., accelerometers, gyroscopes and magnetometers) are used to capture simple and complex activities patterns that cover nearly all aspects of human motions by multiple subjects.

The USC-HAD dataset [30] is taken from a motion node device which includes a wearable network of 6-Degrees of freedom (DoF) for sensing and 3D motion tracking. It consists of multiple sensors such as a gyroscope and an accelerometer, which give real time orientations. These sensors are located at the front right hip because it is one of the top five locations used in [31]. A group of 14 subjects performed 12 different activities, namely, jumping up, running forward, walking forward, elevator down, elevator up, sitting, standing, sleeping, walking left, walking right, walking downstairs and walking upstairs. The devices used in this experiment have a sampling rate of 100 Hz.

The second dataset is our proposed self-annotated IM-sporting behavior (IMSB) dataset [32], which embodies data from three body worn accelerometers. These sensors are located at the knee and below the neck and wrist regions to capture different aspects of human motions. Group of 20 subjects performed six different sporting behaviors, namely, football, skipping, basketball, badminton, cycling, and table tennis. The volunteers were both professionals and athletes. The age range of the volunteers was 20–30 years-old and their weight range was 60–100 kgs. The experimental environments involved indoor/outdoor courts to record different motions of athletes in different situations.

We also tested with a third benchmark dataset named Mhealth. The Mhealth dataset [33] includes both static and dynamic activities of ten subjects, which are recorded from different sensors such as one 2-lead Electrocardiogram (ECG) sensor, two 3-axis gyroscope sensors, three 3-axis magnetometer sensors, and three 3-axis accelerometer sensors. These sensors are located at the left ankle, the right wrist and the chest. This dataset includes twelve different outdoor activities, namely, standing still, lying down, sitting and relaxing, walking, jogging, running, cycling, climbing stairs, crouching, waist bends forward, jump front and back, and frontal elevation of arms.

### 5.2. Hardware Platform

**Figure 12:**
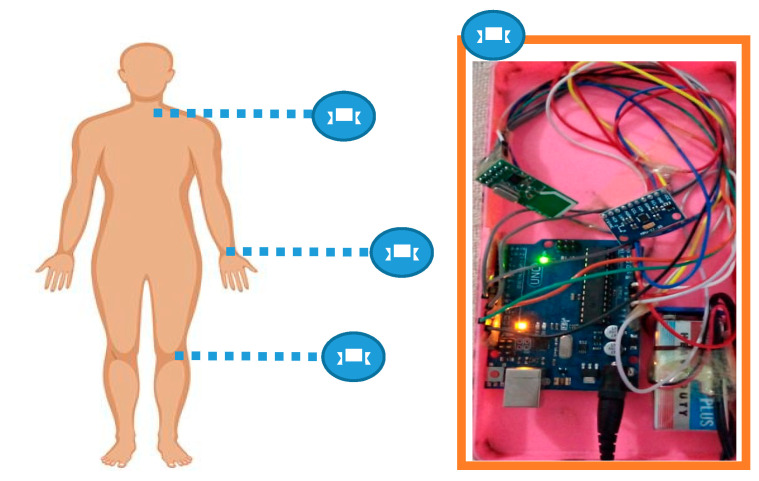
Sensors mounted on human body.

In the experiment of the HAR system, the sensor platform comprised of three MPU6050 sensors (InvenSense, San Jose, CA, USA). These sensors were interfaced with the Arduino device using jumper wires for electrical communication. Three NRF24L01 (Nordic Semiconductor, Trondheim, Norway) modules were also connected with the MPU6050 sensors. All three modules were responsible for the transmission of data to the fourth module. The setup of the fourth module, known as the receiver module, was completed by connecting the Arduino (Smart Projects, Italy), NRFL01 (Spark Fun Electronics, Boulder, USA) and a memory card. At each instance of data collection, three modules were mounted at the wrist, knee and below the neck, as shown in Figure 12. The fourth module was connected with the computer, which receives data from three sensors mounted on human body. The 9-volt batteries were used with the setup to obtain uninterrupted data wirelessly. The open source Arduino software (IDE) was used to simulate the operation in a real-time environment.

The MPU6050 sensor module is a complete 6-axis “motion tracking device”. It has a 3-axis gyroscope, a 3-axis accelerometer and a “digital motion processor” combined in a small package and based on micro electro mechanical system (MEMS) technology. The advantage of the MPU6050 sensor is its in-built digital motion processor (DMP) which is used for the computation of motion processing protocols. We received signal data of angles of yaw, roll and pitch. Thus, the effort of the host in the computation of the motion data is minimized.

With the current setup having 9-volt battery, the lifetime of the setup can operate up to 30 h, or less than two days. Therefore, it is recommended to recharge or replace the battery, so that sensors can fulfill their role for longer in the HAR system.

### 5.3. Experimental Result and Evaluation 

In this section, experiments are repeated twice to evaluate the performance of the proposed wearable HAR model compared to the three benchmark datasets. Table 1 depicts the confusion matrix of human activity recognition of 12 different activities using the USC-HAD dataset with a mean accuracy of 91.25% using the 6-observations problem. On the other hand, Table 2 presents the recognition results on the IMSB dataset with a mean accuracy of 93.6% using 3-observations. Table 3 shows the confusion matrix of 12 different physical outdoor activities with a mean accuracy of 90.91% using the Mhealth dataset.

**Figure 13:**
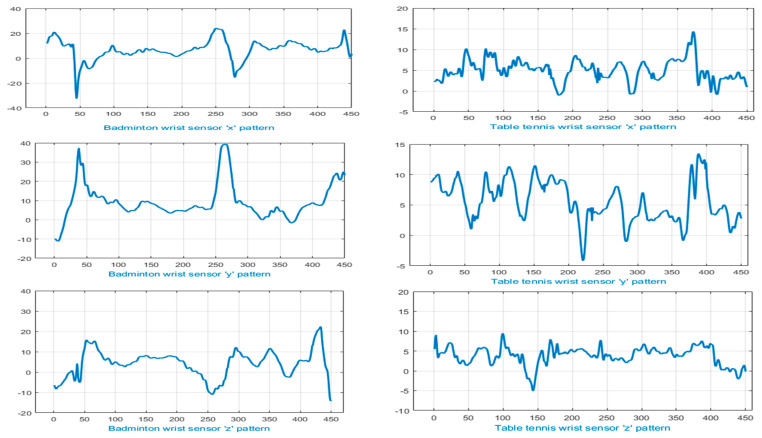
Signals representing wrist motion in badminton (left column) and table tennis (right column).

**Figure 14:**
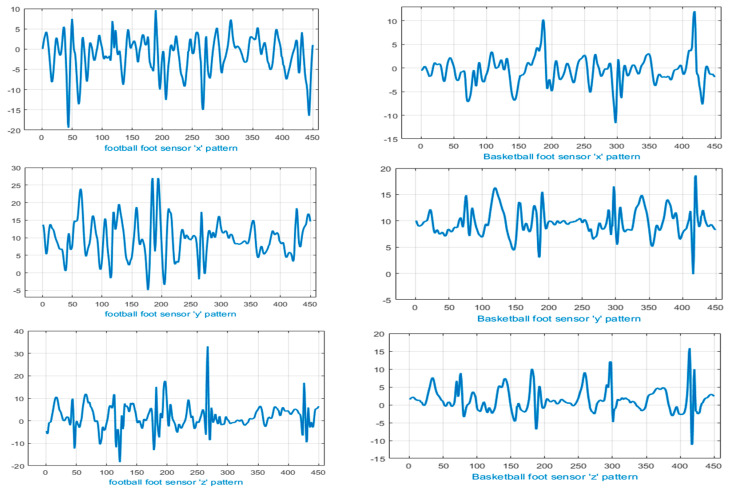
Signals representing feet movement in football (left column) and basketball (right column).

From Figure 13 and Figure 14, it can be observed that a few sports activity pairs, i.e., badminton and table tennis and football and basketball involve high resemblance in motion patterns, i.e., forehand smashing, split-step footwork, defending, rushing and jumping. Therefore, our proposed multifused wearable HAR model highlighted uniqueness factors of badminton and table tennis by recognizing specific movements of wrist motion (see Figure 13). Similarly, Figure 14 shows segregated patterns of feet movements in the cases of football and basketball using the IMSB dataset.

In Table 4, Table 5 and Table 6, we present the comparative study of the proposed model with two other statistically well-known classifiers, i.e., random forest and artificial neural network (ANN) classifiers using precision, recall and F-measure parameters. Overall results show that the proposed method achieved significantly far better performance than the other classifiers. Table 7 shows the comparison results using USC-HAD, IMSB, and Mhealth datasets, respectively.

Finally, our proposed model faces the following certain challenges in practical implementation:

- In practical implementation, we faced pattern issues of human motions of the same activities being performed by different subjects.
- Wearable sensors are highly sensitive in terms of orientation and positions of the subject’s body, and therefore, data readings can be quite different if the sensor is placed slightly above or below the exact locations (i.e., the wrist, knee and below the neck).

## 6. Conclusions

In this paper, we have presented a robust framework that can precisely predict HAR of two challenging datasets in a multisensor environment by catering the augmented signal via multifused features. These features include statistical properties, 1D-LBP, CZT, WHT and first order derivative features to extract the optimal data. In addition, Adam and AdaDelta are used to optimize, train and recognize different types of daily life log and sporting activities. Our proposed system outperforms the others in term of accuracy and shows 91.25%, 90.91, and 93.66% improved results when compared with USC-HAD, IMSB and Mhealth datasets, respectively.

In the future, we will adapt new feature extraction strategies from other domains to classify much more complex activities of different scenarios such as the smart home, offices and public malls via other advanced wearable sensors. In addition, we plan to introduce elderly people to our setups at homes and hospitals.

## Figures and Tables

**Table 1:** Confusion matrix of human activity recognition (HAR) accuracies of individual activities using the USC-HAD dataset.

Activities	A1	A2	A3	A4	A5	A6	A7	A8	A9	A10	A11	A12
**A1**	**0.92**	0.05	0	0	0.02	0	0	0	0.01	0	0	0
**A2**	0.05	**0.89**	0	0	0	0	0.04	0	0	0	0.02	0
**A3**	0	0	**0.96**	0	0	0	0.02	0.02	0	0	0	0
**A4**	0.04	0.01	0	**0.92**	0	0	0.03	0	0	0	0	0
**A5**	0	0.03	0	0	**0.94**	0	0	0	0	0.02	0	0.01
**A6**	0	0	0.02	0	0.02	**0.91**	0	0.02	0	0	0.03	0
**A7**	0	0	0	0.03	0	0.01	**0.95**	0	0	0.01	0	0
**A8**	0.03	0	0.02	0	0	0.03	0	**0.88**	0	0.02	0	0.02
**A9**	0	0.02	0	0.04	0	0	0.02	0.03	**0.86**	0.03	0	0
**A10**	0	0	0	0.03	0	0.02	0	0	0	**0.93**	0.02	0
**A11**	0	0	0.03	0	0	0.05	0	0.02	0.03	0	**0.87**	0
**A12**	0	0	0.03	0	0.01	0	0.01	0	0	0.03	0	**0.92**
**Mean Accuracy = 91.25%**												

A1 = jumping up; A2 = running forward; A3 = walking forward; A4 = elevator down; A5 = elevator up; A6 = sitting; A7 = standing; A8 = sleeping; A9 = walking left; A10 = walking right; A11 = walking downstairs and A12 = walking upstairs. In addition, diagonal values are an outcome, where the model correctly predicts the positive class.

**Table 2:** Confusion matrix of HAR accuracies of individual activities using the IMSB dataset.

Activity	S1	S2	S3	S4	S5	S6
**S1**	**0.93**	0	0.07	0	0	0
**S2**	0	**0.96**	0.04	0	0	0
**S3**	0.06	0	**0.94**	0	0	0
**S4**	0.02	0	0	**0.89**	0	0.09
**S5**	0	0.02	0.02	0	**0.96**	0
**S6**	0	0	0.01	0.05	0	**0.94**
**Mean Accuracy = 93.6%**						

S1 = football; S2 = skipping; S3 = basketball; S4 = badminton; S5 = cycling and S6 = table tennis. In addition, diagonal values are an outcome, where the model correctly predicts the positive class.

**Table 3:** Confusion matrix of HAR accuracies of individual activities using the Mhealth dataset.

Activities	H1	H2	H3	H4	H5	H6	H7	H8	H9	H10	H11	H12
**H1**	**0.94**	0	0	0	0.02	0.03	0	0	0.01	0.01	0	0.01
**H2**	0.04	**0.90**	0.02	0.01	0	0	0.01	0	0	0	0.01	0.01
**H3**	0.01	0.04	**0.93**	0	0	0	0	0.01	0	0	0	0.01
**H4**	0	0	0.02	**0.96**	0	0.01	0	0	0	0	0.01	0
**H5**	0.01	0.04	0	0	**0.92**	0.02	0	0	0	0	0.02	0
**H6**	0.01	0	0	0.02	0.03	**0.94**	0	0.01	0	0	0	0
**H7**	0.02	0	0	0.02	0.02	0.01	**0.89**	0.02	0	0	0.02	0
**H8**	0	0.02	0.01	0	0	0	0.03	**0.91**	0	0.02	0	0
**H9**	0	0.02	0	0.03	0	0	0.03	0.02	**0.87**	0.02	0.01	0
**H10**	0.01	0.02	0	0.01	0	0.03	0	0.01	0	**0.90**	0.01	0.01
**H11**	0	0.02	0	0	0	0.03	0.01	0	0	0.02	**0.91**	0.01
**H12**	0.01	0.03	0.02	0.02	0	0	0.03	0	0	0.04	0	**0.84**
**Mean Accuracy = 90.91%**												

H1 = standing still; H2 = lying down; H3 = sitting and relaxing; H4 = walking; H5 = jogging; H6 = running; H7 = cycling; H8 = climbing stairs; H9 = crouching; H10 = waist bends forward; H11 = jump front and back; H12 = frontal elevation of arms. In addition, diagonal values are an outcome, where the model correctly predicts the positive class.

**Table 4:** Classification results of the three classifiers using the USC-HAD dataset.

Methods	Maximum Entropy Markov Model			Random Forest			ANN		
Activities	Precision	Recall	F-Measure	Precision	Recall	F-Measure	Precision	Recall	F-Measure
**A1**	0.821	0.730	0.773	0.808	0.726	0.764	0.806	0.724	0.763
**A2**	0.816	0.723	0.767	0.798	0.719	0.756	0.794	0.717	0.753
**A3**	0.827	0.738	0.780	0.858	0.765	0.808	0.853	0.732	0.788
**A4**	0.821	0.730	0.773	0.712	0.641	0.674	0.704	0.724	0.714
**A5**	0.824	0.734	0.776	0.828	0.748	0.785	0.821	0.728	0.772
**A6**	0.819	0.728	0.771	0.812	0.738	0.773	0.805	0.722	0.761
**A7**	0.826	0.736	0.778	0.725	0.668	0.695	0.715	0.730	0.722
**A8**	0.814	0.721	0.765	0.718	0.658	0.686	0.711	0.715	0.713
**A9**	0.811	0.716	0.761	0.762	0.693	0.725	0.756	0.710	0.732
**A10**	0.823	0.732	0.775	0.871	0.717	0.786	0.866	0.726	0.790
**A11**	0.813	0.719	0.763	0.796	0.709	0.749	0.783	0.713	0.746
**A12**	0.821	0.730	0.773	0.807	0.724	0.763	0.804	0.723	0.761

**Table 5:** Classification results of the three classifiers using the IMSB dataset.

Dynamic Activities	Maximum Entropy Markov Model			Random Forest			ANN		
Activities	Precision	Recall	F-Measure	Precision	Recall	F-Measure	Precision	Recall	F-Measure
**S1**	0.877	0.892	0.884	0.868	0.836	0.851	0.864	0.833	0.848
**S2**	0.867	0.884	0.876	0.848	0.832	0.839	0.841	0.821	0.831
**S3**	0.870	0.886	0.878	0.865	0.827	0.845	0.861	0.824	0.842
**S4**	0.858	0.876	0.867	0.847	0.801	0.822	0.837	0.809	0.823
**S5**	0.866	0.883	0.875	0.864	0.823	0.842	0.855	0.819	0.837
**S6**	0.877	0.892	0.884	0.869	0.833	0.850	0.858	0.833	0.845

**Table 6:** Classification results of the three classifiers using the Mhealth dataset.

Methods	Maximum Entropy Markov Model			Random Forest			ANN		
Activities	Precision	Recall	F-Measure	Precision	Recall	F-Measure	Precision	Recall	F-Measure
**H1**	0.728	0.691	0.709	0.712	0.676	0.693	0.706	0.671	0.688
**H2**	0.720	0.681	0.700	0.703	0.666	0.684	0.697	0.661	0.679
**H3**	0.726	0.688	0.707	0.709	0.673	0.691	0.704	0.669	0.686
**H4**	0.732	0.695	0.713	0.716	0.680	0.698	0.711	0.676	0.693
**H5**	0.724	0.686	0.704	0.707	0.671	0.689	0.702	0.666	0.684
**H6**	0.728	0.691	0.709	0.712	0.676	0.693	0.706	0.671	0.688
**H7**	0.717	0.679	0.698	0.701	0.664	0.681	0.695	0.659	0.676
**H8**	0.722	0.684	0.702	0.705	0.669	0.686	0.700	0.664	0.681
**H9**	0.713	0.674	0.693	0.696	0.659	0.677	0.690	0.654	0.671
**H10**	0.720	0.681	0.700	0.703	0.667	0.684	0.697	0.661	0.679
**H11**	0.722	0.684	0.702	0.705	0.669	0.686	0.700	0.664	0.681
**H12**	0.705	0.666	0.685	0.688	0.651	0.669	0.682	0.646	0.664

**Table 7:** Comparison of the proposed method’s accuracy with state-of-the-art methods using the USC-HAD, IMSB and Mhealth datasets.

Methods	Recognition Accuracy using USC-HAD (%)	Recognition Accuracy using IMSB (%)	Recognition Accuracy using Mhealth(%)
Classification using Random Forest [34]	90.7	85.43	-
Classification using Single Layer [35]	89.3	-	-
Ensemble algorithms [36,37]	86.9	-	90.01
Classification using LSVM [38]	-	80	-
Hampel Estimated Module [39]	-	-	85.18
Symbolic approximation [40]	84.3	-	-
Proposed HAR + Ada Delta	**90.79**	**90.13**	**88.67**
Proposed HAR + Adam	**91.25**	**93.66**	**90.91**

Bold letters for proposed recognition accuracy.
